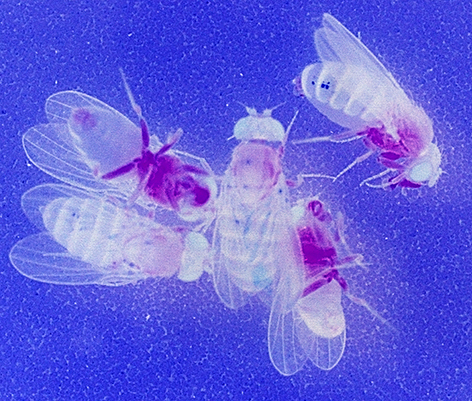# High-throughput drug screening in model systems: lessons from zebrafish and *Drosophila*

**Published:** 2014-11

Automated, rapid – high-throughput – systems are an emerging and successful strategy to optimise drug screening and search for new therapeutics. This approach is even more effective when performed on model organisms that can be easily manipulated experimentally and have features that mimic those of human diseases. An example is the work by Carol Kim and colleagues, who developed a zebrafish model of human influenza. Influenza A viruses (IAVs) are an important health concern owing to the risk of global pandemics and the high rate of virus mutation, which can give rise to new antiviral-resistant strains. Here, the authors infected zebrafish embryos with human IAVs and demonstrated that this model could recapitulate several features of human influenza infection, including a heightened innate immune response and typical pathological alterations such as edema and tissue destruction. By using a fluorescent reporter strain of IAV, the authors were also able to visualise IAV infection *in vivo*. In addition, administration of a known antiviral compound significantly reduced mortality in infected zebrafish. Therefore, the zebrafish represents an exquisite system to model human IAV infection, allowing the study of host-pathogen interactions in real time and high-throughput validation of novel anti-influenza drugs. **Page 1227**

**Figure:**
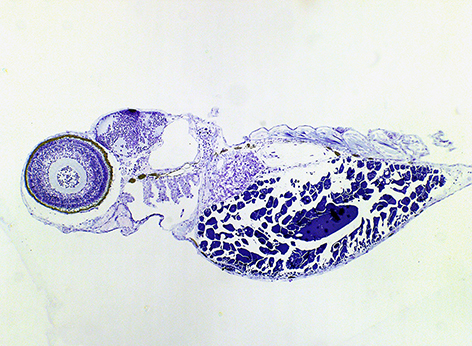


Flies are another extremely useful system for *in vivo* pharmacological screening, as demonstrated in the study by Arturo López Castel and colleagues. This group used *Drosophila* as an experimental platform to investigate mechanisms of myotonic dystrophy type 1 (DM1) and search for potential anti-disease compounds. DM1 is a genetic neuromuscular disorder caused by spliceopathy: defective regulation of mRNA alternative splicing. In this disease, mutant mRNAs, containing expanded CUG trinucleotide repeats, are thought to interact with key splicing modulators and lead to alternative splicing misregulation. The authors used DM1 transgenic flies expressing a luciferase splicing reporter system, in which the expression of DM1-linked splicing variants could be ‘sensed’ via the read-out of a luminescent signal. These *Drosophila* spliceosensors were grown on a 96-well plate and used as a platform to screen for more than 16,000 small molecules. Among these, several compounds were identified for their ability to reduce aberrant ribonuclear aggregates or bind toxic RNA molecules. This study not only revealed promising drug candidates for DM1, but developed a reliable system that could also be applied to other spliceopathies. **Page 1297**

**Figure:**